# Constructing Hypothetical Risk Data from the Area under the ROC Curve: Modelling Distributions of Polygenic Risk

**DOI:** 10.1371/journal.pone.0152359

**Published:** 2016-03-29

**Authors:** Suman Kundu, Jannigje G. Kers, A. Cecile J. W. Janssens

**Affiliations:** 1 Department of Epidemiology, Rollins School of Public Health, Emory University, Atlanta, GA, USA; 2 Department of Epidemiology, Erasmus University Medical Center, Rotterdam, The Netherlands; 3 Department of Clinical Genetics/EMGO Institute for Health and Care Research, Section Community Genetics, VU University Medical Center, Amsterdam, The Netherlands; Institute for Clinical Epidemiology and Applied Biometry, GERMANY

## Abstract

**Background:**

Modeling studies using hypothetical polygenic risk data can be an efficient tool for investigating the effectiveness of downstream applications such as targeting interventions to risk groups to justify whether empirical investigation is warranted. We investigated the assumptions underlying a method that simulates risk data for specific values of the area under the receiver operating characteristic curve (AUC).

**Methods:**

The simulation method constructs risk data for a hypothetical population based on the population disease risk, and the odds ratios and frequencies of genetic variants. By systematically varying the parameters, we investigated under what conditions AUC values represent unique ROC curves with unique risk distributions for patients and nonpatients, and to what extend risk data can be simulated for precise values of the AUC.

**Results:**

Using larger number of genetic variants each with a modest effect, we observed that the distributions of estimated risks of patients and nonpatients were similar for various combinations of the odds ratios and frequencies of the risk alleles. Simulated ROC curves overlapped empirical curves with the same AUC.

**Conclusions:**

Polygenic risk data can be effectively and efficiently created using a simulation method. This allows to further investigate the potential applications of stratifying interventions on the basis of polygenic risk.

## Introduction

Genetic risk models are increasingly being investigated for their ability to predict the risk of multifactorial diseases. Using polygenic risk models, interventions can be targeted to individuals at higher risk to reduce the burden of the diseases while efficiently allocating healthcare resources [1]. The effectiveness of stratified medicine can be investigated in randomized controlled trials, but this is only opportune when the predictive ability of the polygenic model is already high enough to expect health benefits of stratification [2–4].

The expected health benefits can be estimated in simulation studies using hypothetical risk data to justify whether empirical investigation is warranted or premature. The predictive ability of risk models is generally indicated by the area under the receiver operating characteristic (ROC) curve (AUC), which indicates the degree to which the models can distinguish people who will develop the disease and from those who will not. Several methods are available to estimate the expected AUC of a genetic risk model from the odds ratios and frequencies of the genetic variants included [5]. These can be used to estimate AUC for different effect sizes, frequencies and the number of risk alleles, which all impact AUC [6–10]. We recently demonstrated that simulation methods can accurately reproduce the predictive ability of genetic risk models investigated in empirical data [11]. We also demonstrated that a simulation method can be used to assess the predictive ability of polygenic models when empirical data are not available, for example to assess the expected AUC of to be discovered genetic variants or of commercial tests [12]

When modeling methods simulate risk data to estimate the AUC of a risk model, these data can also be used to investigate downstream questions, such as whether a polygenic risk model is predictive enough to allow for useful stratification of risk groups and even whether risk stratification will be cost-effective [13,14]. Simulation of hypothetical data that mimic empirical data can therewith investigate, in advance, how high the predictive ability of genetic risk models needs to be to yield risk stratification programs that are cost-effective. Answering such questions in hypothetical data is possible when each value of the AUC comes with unique risk distributions irrespective of how many genetic variants are included in the risk models and what the effect sizes and frequencies of their risk alleles are.

**Fig 1:**
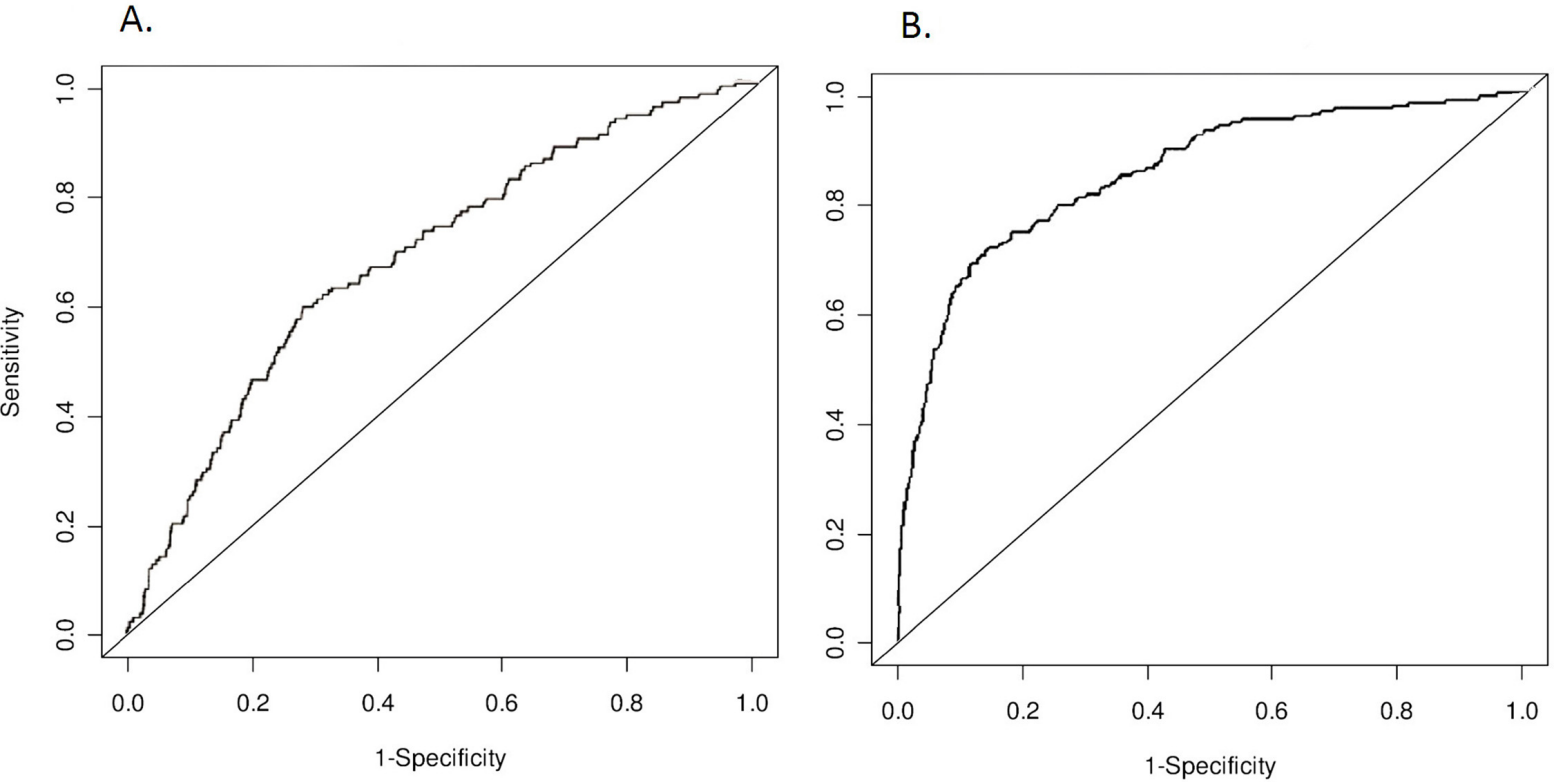
Examples of rounded and non-rounded shapes of receiver operating characteristic (ROC) curves.

From empirical prediction studies, it is clear that ROC plots for different risk models that have the same AUC, typically look the same. ROC curves generally have a ‘rounded’ shape, without a clear angle (**Fig 1A)**. Rounded curves result when risk models include continuous variables or a large number of categorical variables with similar effects on disease risk, such as is seen in polygenic risk models. ROC curves may have an ‘angle’ when one, often binary, predictor has a stronger effect on disease risk than all other variables in the model (**Fig 1B**).

When ROC curves can be assumed to have a rounded shape, several inferences can be made. First, when ROC curves are rounded, the lines of different curves do not cross and hence, they each are represented by a unique AUC value. Second, and vice versa, each AUC value represents a unique ROC curve. Third, unique ROC curves represent unique underlying risk distributions for patients and nonpatients, irrespective of the number of genetic variants in the model, their effect sizes and the frequencies of the risk alleles.

In this study, we investigated the validity of these assumptions that would enable the modeling of risk data from an AUC value. We examined under which conditions: the AUC value can be assumed to represent a unique ROC curve and the ROC curve can be assumed to represent unique risk distributions irrespective of the effect sizes and frequencies of variants that are included in the model. We start by demonstrating that risk distributions can be simulated for precise values of the AUC, which is relevant as small differences in AUC are often found in empirical studies [15]. To illustrate the accuracy of the method we aimed to reproduce the ROC curves of published empirical studies.

## Methods

### Simulation method

The modeling procedure has been described in detail elsewhere [7]. In short, the procedure creates a dataset of individual genotypes and disease status for a hypothetical population. Genotypes are randomly assigned to individuals in such a way that the genotype frequencies matched prespecified values. To assign disease status, we first calculate for each individual the predicted risk of disease based on their multi-locus genotype status, the odds ratio of each variant and the population disease risk using Bayes theorem. Next, disease status is assigned based on a procedure that compares the predicted risk for each individual to a randomly drawn value between 0 and 1 from a uniform distribution. An individual is assigned to develop the disease (patients) when the predicted risk of disease is higher than the random value and to not develop the disease (non-patients) when the predicted risk is lower. The latter strategy implies that the risk model is perfectly calibrated. Predicted risks and disease status were used to calculate the AUC, which was obtained using the package ROCR in R software. We assumed that all SNPs had the same per allele odds ratio and risk allele frequencies, but these were varied between scenarios. Population size was 100,000 in all scenarios.

To obtain the risk data for a specific value of the AUC, we used an iterative procedure in which we added as many genetic variants until the AUC of the prediction model reached a prespecified value. To this end, we calculated predicted risks using Bayes’ theorem, assigned disease status and obtained the AUC of the prediction model after each variant added, as described above. The procedure was stopped when the AUC value exceeded the prespecified value and then the risk distribution for which the AUC value was closest to the prespecified value, was considered. The AUC value, as well as the population disease risk, the odds ratios and frequencies of the risk alleles that were used to construct the risk distributions, were varied between scenarios. The codes to make a risk dataset for a specific value of the AUC were provided in S1 File.

### Data analysis

First, we examined whether we can accurately simulate risk data for a specific value of the AUC. As the addition of genetic variants to prediction models often increases the AUC only minimally, we need to model risk data for precise values of the AUC if we wish to investigate such scenarios in hypothetical data. To investigate the precision of the modeling, we simulated 100 risk datasets for a specific AUC value and counted the number of times the observed AUC value was within a range from the specified value. We considered ranges of 0.005 and 0.01. We recorded the number of SNPs that were used in the modelling. We investigated three different values of AUC (0.60, 0.70 and 0.80) and varied the per allele odds ratios (ranging from 1.1 to 2.0) and the frequencies (10% and 30%) of the risk alleles.

Second, to investigate whether the AUC value can be assumed to represent a unique ROC curve, which is the case when the curves have a rounded shape, we examined under what conditions this assumption holds. Non-rounded shapes typically occur when one binary risk factor, or one category of a categorical variable, has a stronger impact on the disease risk than the rest. Therefore, and because we were only interested in the approximate odds ratio that changes the shape of the curve, we investigated the odds ratio that is needed to visibly change the rounded shape of ROC curves of prediction models with AUC values of 0.60, 0.70 and 0.80. We first constructed perfectly rounded shapes by modelling risk factors that all had the same low odds ratio of 1.1 and risk allele frequency of 30%. These curves will be referred to as reference curves. We constructed a ‘band’ around this reference line by plotting two ROC curves so that the delta AUC form the reference ROC curve was 0.02 in either direction. We considered ROC curves to have a rounded shape when the entire curve was within this band. We then constructed risk models in which we increased the odds ratio of one binary predictor between scenarios. To this risk factor, we added as many other risk factors with low odds ratios of 1.1 to achieve the specified AUC. We increased the odds ratio of the binary risk factor until the shape of the ROC curve was no longer rounded. We present examples of ROC curves that are inside and just outside the band and report the odds ratio of the binary risk factor. The frequency of the binary risk factor was 5% and the population disease risk was 20%.

Third, to investigate whether a specific ROC curve results from unique underlying risk distributions, we examined whether risk distributions for the same AUC value are similar when they are modelled using different combinations of odds ratios and frequencies of genetic variants. Because we model the same odds ratios for all genetic variants, the ROC curves will all be rounded. For each combination of the parameters (AUC, odds ratios and risk allele frequencies), we simulated 100 datasets and calculated the average of the mean, standard deviation, and the 5th, 25^th^, 50^th^, 75^th^ and 95^th^ percentiles of the risk distributions in patients and non-patients separately. AUC values were 0.60, 0.70 and 0.80, odds ratios ranged between 1.05 and 1.40, and risk allele frequencies were 10, 20 and 30 percent. The population disease risk was 20%. We simulated 100 datasets for which the observed AUC value of the risk model was within 0.005 of the pre-specified value.

Finally, the method was illustrated by replicating published ROC curves and risk distributions. ROC plots and risk distributions were randomly selected from the literature. We randomly selected ROC plots from empirical studies with sufficient sample size (n>1,000).

## Results

Table 1 shows how accurately risk data can be modelled for precise values of the AUC. All simulation attempts to create risk data for which the observed AUC was within 0.01 of the specified AUC (0.60, 0.70 or 0.80) were successful. When we attempted to model risk data for which the AUC was less than 0.005 from the specified AUC, we were only successful in all attempts when the number of genetic variants was sufficiently large. For example, all 100 simulations in which we modelled variants with an odds ratio of 2.0 and risk allele frequencies of 10% to produce an AUC of 0.70 had observed AUCs that were between 0.690 and 0.710, but only 75% of the simulations had an observed AUC between 0.695 and 0.705. These findings suggest that the odds ratios need to be set low enough so that the risk model is constructed using a large number of genetic variants.

**Table 1:** Accuracy of observed AUC values for different combinations of odds ratios and frequencies of the risk alleles.

AUC	Frequency (%)	OR	Percentage of simulated AUCs within range		Number of SNPs in 100 simulations	
			AUC ± 0.01	AUC ± 0.005	Mean	SD
0.60	10	1.1	100	100	78	3.6
		1.2	100	100	21	0.9
		1.5	100	63	5	0.4
	30	1.05	100	100	130	6.4
		1.1	100	100	34	1.8
		1.2	100	97	9	0.5
0.70	10	1.2	100	100	98	2.3
		1.5	100	98	19	0.6
		2.0	100	75	7	0.2
	30	1.1	100	100	157	4.0
		1.2	100	100	42	1.2
		1.5	100	80	9	0.4
0.80	10	1.5	100	100	58	1.1
		2.0	100	100	19	0.5
		3.0	100	69	8	0.17
	30	1.2	100	100	127	2.4
		1.5	100	99	25	0.6
		2.0	100	78	9	0.3

Legend: AUC = area under receiver operating characteristic curve; OR = odds ratio; SNPs = single nucleotide polymorphisms.

**Fig 2:**
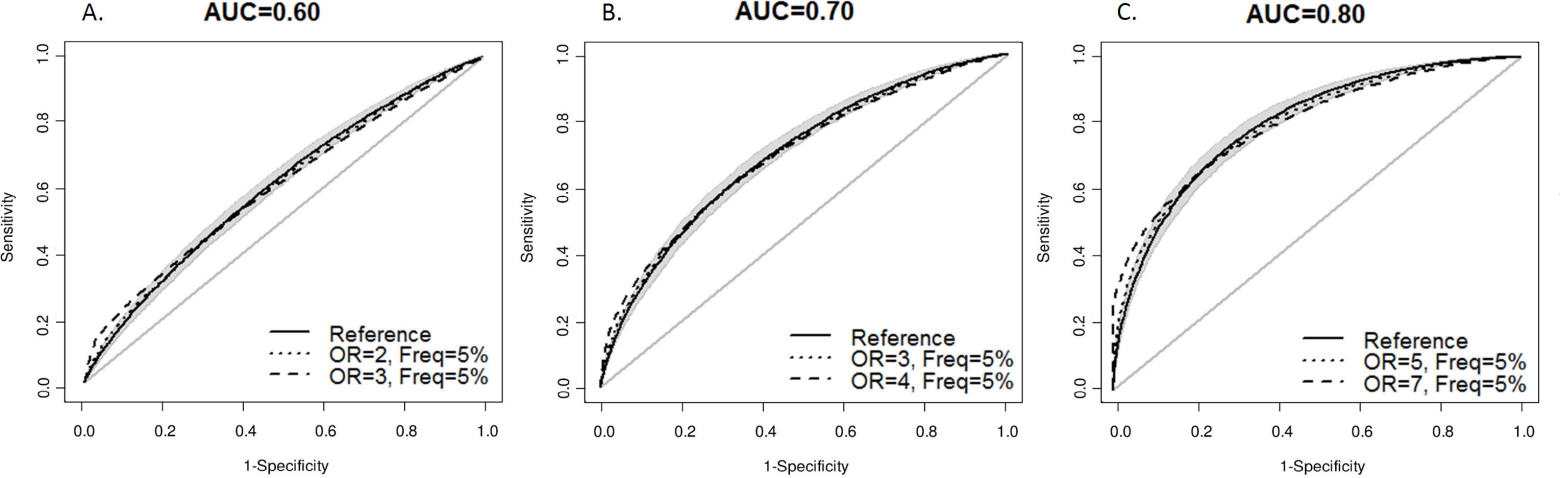
Odds ratios needed to produce a receiver operating characteristic (ROC) curve that did not have a rounded shape. See methods for definition of rounded shape. AUC = area under receiver operating characteristic curve, OR = odds ratio, Freq = frequency. The odds ratio and frequency refer to the single binary variable in the risk model.

Fig 2 shows rounded ROC curves for prediction models that were based on susceptibility variants with OR of 1.10 only (reference line), as well as ROC curves for prediction models in which one binary predictor was modelled to have a stronger effect to produce a curve that would no longer have a rounded shape (Fig 2). The figures show that a strong predictor was needed to produce an ROC curve that was no longer considered to be rounded; for example, when the AUC of 0.70 the added variant needed to have an odds ratio of 4 to yield a ROC curve that was considered not rounded.

**Fig 3:**
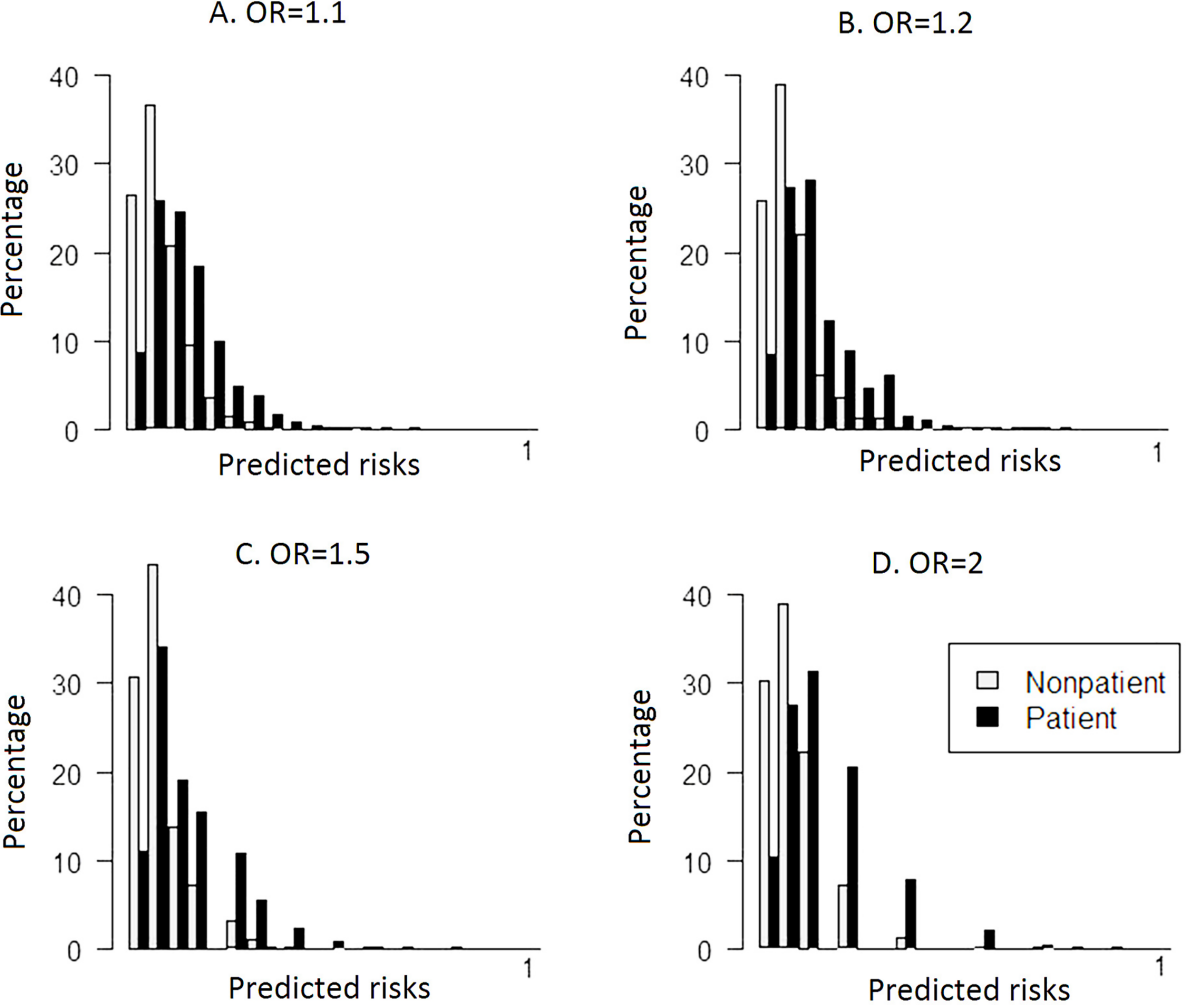
Histograms of predicted risks of patients and nonpatients for scenarios with varying odds ratios. The area under the receiver operating characteristic curve was 0.70 and risk allele frequencies were 30%. OR = odds ratio.

Table 2 shows that different combinations of odds ratios and risk allele frequencies yielded the same risk distributions for patients and non-patients for each value of the AUC. The mean, median and percentiles were similar up to two decimals with corresponding standard deviations equal or close to zero. Fig 3 shows distributions of predicted risks of patients and non-patients for four scenarios with varied ORs that yielded the same AUC of 0.70 (Fig 3). These graphs show that the distributions were identical when the number of SNPs was sufficiently large (154 and 48 in Fig 3A and 3B), irrespective of the magnitude of the odds ratio. Findings were similar for the distributions of unweighted risk scores (**S1 Fig**).

**Table 2:** Characteristics of risk distributions for patients and nonpatients constructed modelled from different combinations of per allele odds ratios and risk allele frequencies.

	SNPs			Patients							Nonpatients						
AUC	OR	f (%)	n	Mean	SD	Percentile					Mean	SD	Percentile				
						5^th^	25^th^	50^th^	75^th^	95^th^			5^th^	25^th^	50^th^	75^th^	95^th^
0.60	1.05	10	290	0.22	0.06	0.13	0.17	0.21	0.25	0.33	0.20	0.06	0.12	0.16	0.19	0.23	0.30
		20	166	0.22	0.06	0.13	0.17	0.21	0.25	0.32	0.20	0.06	0.12	0.16	0.19	0.23	0.30
		30	126	0.22	0.06	0.13	0.17	0.21	0.25	0.32	0.20	0.06	0.12	0.16	0.19	0.23	0.30
	1.1	10	75	0.22	0.06	0.13	0.17	0.21	0.25	0.33	0.20	0.06	0.12	0.16	0.19	0.23	0.30
		20	43	0.22	0.06	0.13	0.17	0.21	0.25	0.33	0.20	0.06	0.12	0.15	0.19	0.23	0.30
		30	33	0.22	0.06	0.13	0.17	0.21	0.25	0.32	0.20	0.06	0.12	0.16	0.19	0.23	0.30
0.70	1.1	10	354	0.27	0.14	0.09	0.17	0.25	0.35	0.53	0.18	0.11	0.06	0.10	0.16	0.24	0.39
		20	201	0.27	0.14	0.09	0.17	0.25	0.35	0.53	0.18	0.11	0.05	0.10	0.16	0.24	0.39
		30	154	0.27	0.13	0.09	0.17	0.25	0.35	0.52	0.18	0.11	0.05	0.10	0.16	0.24	0.39
	1.2	10	96	0.27	0.14	0.09	0.17	0.25	0.35	0.54	0.18	0.11	0.06	0.11	0.16	0.23	0.39
		20	54	0.27	0.14	0.09	0.17	0.25	0.36	0.53	0.18	0.11	0.06	0.10	0.16	0.24	0.40
		30	42	0.27	0.14	0.09	0.16	0.25	0.35	0.52	0.18	0.11	0.06	0.10	0.16	0.23	0.39
0.80	1.2	10	289	0.37	0.22	0.07	0.18	0.34	0.53	0.78	0.16	0.14	0.02	0.05	0.11	0.21	0.47
		20	163	0.37	0.22	0.07	0.19	0.33	0.53	0.77	0.16	0.14	0.02	0.05	0.11	0.22	0.46
		30	125	0.37	0.22	0.07	0.19	0.34	0.52	0.77	0.16	0.14	0.02	0.05	0.11	0.22	0.46
	1.3	10	138	0.37	0.22	0.07	0.19	0.34	0.53	0.78	0.16	0.14	0.02	0.05	0.11	0.21	0.47
		20	79	0.37	0.22	0.07	0.18	0.33	0.52	0.78	0.16	0.14	0.02	0.05	0.11	0.22	0.47
		30	61	0.37	0.22	0.07	0.19	0.35	0.54	0.78	0.16	0.15	0.02	0.05	0.11	0.21	0.47
	1.4	10	83	0.37	0.22	0.06	0.19	0.34	0.54	0.79	0.16	0.14	0.02	0.05	0.12	0.21	0.47
		20	48	0.37	0.22	0.07	0.19	0.35	0.53	0.78	0.16	0.15	0.02	0.05	0.11	0.22	0.47
		30	37	0.37	0.22	0.07	0.18	0.36	0.53	0.77	0.16	0.15	0.02	0.05	0.10	0.22	0.46

Legend: SNPs = single nucleotide polymorphisms; AUC = area under receiver operating characteristic curve; OR = odds ratio; f = frequency; n = number of SNPs. All values are means across 100 simulations. Standard deviations of the mean and SD were zero, standard deviations for the percentiles were mostly zero (48%), 0.01 (39%) or 0.02 (12%).

**Fig 4:**
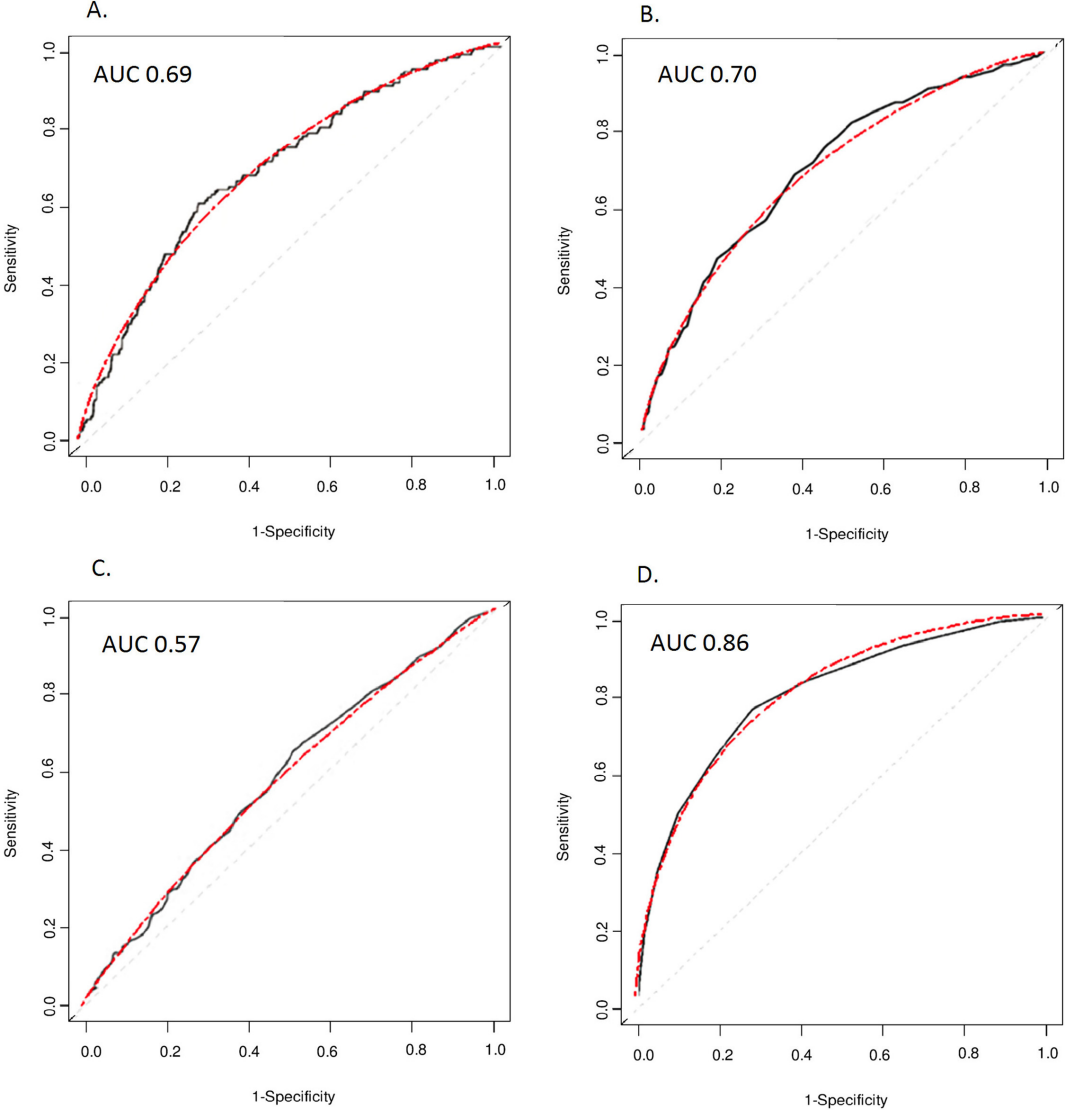
Receiver operating characteristic (ROC) curves from empirical prediction studies and from simulated data based on their AUC values. The ROC curves in black were obtained from the cited articles, the red ones are based on simulated data for the same value of the area under the ROC curve (AUC; A. 0.69 [20]; B. 0.70 [21]; C. 0.57 [22]; D. 0.86 [23]). The lines were pasted in the (empty) plots anchoring the beginning and end of the line at (0,0) and (1,1).

Fig 4 shows the accuracy of ROC curves that were constructed by the simulation method based on AUC and population disease risk only (Fig 4). The ROC curves based on hypothetical risk data were similar to the curves that were published in the empirical prediction studies.

## Discussion

In this study, we showed that risk data can be constructed for specific values of the AUC and the population disease risk. Assuming that ROC curves typically have rounded shapes, we showed that unique risk distributions can be constructed irrespective of the exact values of the parameters on which the modeling is based, namely the number, the odds ratios and frequency of the genetic variants. When the number of variants is sufficiently large, all simulations yielded the same risk distributions for the same value of the AUC. We also showed that risk data can be constructed for very precise values of the AUC.

Before discussing the implications of our findings, two methodological aspects need to be addressed. First, we observed that risk distributions and ROC curves are similar irrespective of the odds ratios and allele frequencies when the number of genetic variants was high. It is not possible to specify when the number was high enough, as this depends on the variable frequencies. Table 2 shows that the risk distributions were similar when the number was at least 30–40, but a higher number may be preferred. Second, we arbitrarily defined that the shape of the ROC curve was no longer rounded when at least one point of the curve crossed a band that was delineated by rounded curves with an AUC value that were 0.02 higher and lower. Different definitions will yield different values of the odds ratios that cause the curve to be non-rounded. The value of the odds ratio also differs with the frequency of the binary risk factor that was added to the model: a higher frequency yields an angle more towards the middle of the ROC curve. The latter needs a higher odds ratio to yield a non-rounded curve, also because the band is wider in the middle.

The simulation method works because the value of the AUC represents the degree of overlap between the risk distributions of patients and non-patients. When the shapes and location of the distributions are the same across different scenarios, the AUC value will be the same, and hence the AUC values can be considered to represent unique underlying risk distributions. The shapes of the distributions follow from the way how we constructed the risk models, in this case by combining a sufficiently large number of variants that all had the same effect on disease risk. When the number of genetic variants is sufficiently large, the distributions of patients and non-patients look the same across different scenarios where the values of the parameters are varied, and hence the AUC values are the same.

To simulate risk data from an AUC value, we departed from the assumption that ROC curves are rounded and hence that each AUC value represented a unique ROC curve and unique risk distributions for patients and non-patients. The essence of the assumption of rounded curves is that the curves of different AUC do not cross. Evidently, for the latter the curves do not need to be rounded per se. The method can also be used when one or more predictors are known to have a much stronger effect on disease risk than other known and unknown variables. In this scenario, such as is the case for age-related macular degeneration and Crohn’s disease, the known predictors can be modelled with their effect sizes and the model can be supplemented by other predictors with small effect sizes (e.g., 1.1) to achieve an AUC of specified value.

We used a simulation approach to construct risk data for a certain AUC value in which we created risk models by adding as many genetic variants to achieve a certain AUC. It should be noted that risk data can also be modelled by assuming shapes of risk distributions and specifying means and standard deviations for patients and non-patients [16].

We choose a simulation approach because we used a method that was initially developed and primarily used to investigate the predictive ability and utility of polygenic risk models [7]. We therefore preferred an intuitive strategy that is similar to adding genetic variants to an empirical risk model.

The simulation method was designed to model data from polygenic risk models, but its application is not limited to that. We showed that the method yields similar risk distributions irrespective of what the values of the parameters, the number of genetic variants, their odds ratios and frequencies, are. Risk distributions will be similar when continuous risk factors are included in the model, when there is correlation between the predictors or when interaction effects are included. It is the value of the AUC that determines the shape of the risk distributions.

Simulating risk distributions of patients and non-patients can be used to investigate downstream applications such as the effectiveness of stratified medicine [13,14]. Simulation studies can specify under what conditions stratified medicine, giving different treatments to different risk groups, can be more beneficial than population-wide programs in which everyone receives the same treatment [17,18]. For example, given certain effectiveness of interventions, simulation studies can inform how predictive risks models need to be for stratification to be more effective and efficient than population-wide programs. Such questions are relevant e.g., to improve the efficiency and effectiveness of cancer screening [19]. Investigations in simulated data can inform the debate on the promise of precision medicine, and be useful to quantify the health benefits when empirical data are not available.

## Supporting Information

S1 Fig Histograms of unweighted risk scores of patients and nonpatients. The area under the receiver operating characteristic curve was 0.70.
(TIFF)

S1 File R code for the simulation of the risk data based on AUC value. (TXT)
