# Supplementary figures and images for: Constructing Hypothetical Risk Data from the Area under the ROC Curve: Modelling Distributions of Polygenic Risk
Source: PLoS One. 2016 Mar 29;11(3):e0152359. doi: 10.1371/journal.pone.0152359 (PMC4811433; doi:10.1371/journal.pone.0152359)

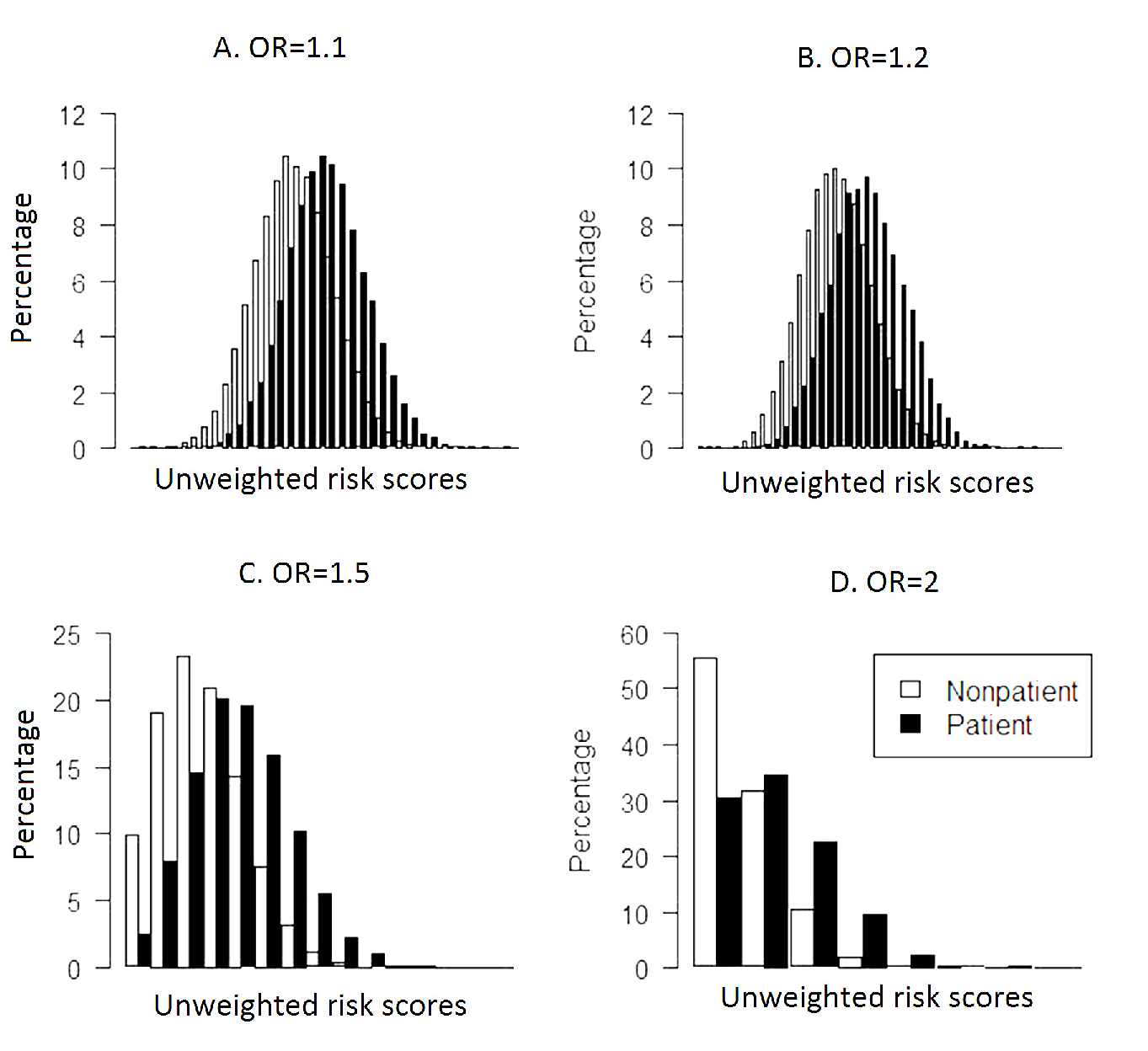

Supplement: S1 Fig — The area under the receiver operating characteristic curve was 0.70. (TIFF) [file pone.0152359.s001.tiff]
